# Merkel Cell Polyomavirus Strains in Patients with Merkel Cell Carcinoma

**DOI:** 10.3201/eid1506.081463

**Published:** 2009-06

**Authors:** Antoine Touzé, Julien Gaitan, Annabel Maruani, Emmanuelle Le Bidre, Angélique Doussinaud, Christine Clavel, Anne Durlach, François Aubin, Serge Guyétant, Gérard Lorette, Pierre Coursaget

**Affiliations:** Institut National de la Santé et de la Recherche Médicale (INSERM) Unit 618, Tours, France (A. Touzé, J. Gaitan, A. Doussinaud, P. Coursaget); Université François Rabelais, Tours (A. Touzé, J. Gaitan, A. Doussinaud, S. Guyétant, G. Lorette, P. Coursaget, A. Maruani, E. Le Bidre); Centre Hospitalier Régional Universitaire (CHRU)–Trousseau, Tours (A. Maruani, E. Le Bidre, S. Guyétant, G. Lorette); INSERM Unit 903, Reims, France (C. Clavel, A. Durlach); CHRU de Reims (C. Clavel, A. Durlach); Université de Franche–Comté, Hôpital Saint-Jacques, Besançon, France (F. Aubin)

**Keywords:** Carcinoma, Merkel cells, virus transforming agent, polyomavirus, viruses, France, dispatch

## Abstract

We investigated whether Merkel cell carcinoma (MCC) patients in France carry Merkel cell polyomavirus (MCPyV) and then identified strain variations. All frozen MCC specimens and 45% of formalin-fixed and paraffin-embedded specimens, but none of the non-MCC neuroendocrine carcinomas specimens, had MCPyV. Strains from France and the United States were similar.

Although infectious agents have been recognized as etiologic agents in ≈20% of cancers (*1*), the list of oncogenic infectious agents is limited. A new virus, Merkel cell polyomavirus (MCPyV), recently was discovered in humans with Merkel cell carcinoma (MCC), a relatively rare, aggressive primary cutaneous neuroendocrine carcinoma. Feng et al. (*2*) reported PCR detection of MCPyV in most MCC specimens (*2*), and clonal integration of the viral genome has been identified.

Polyomaviruses are small nonenveloped DNA viruses, with a double-stranded circular DNA genome of ≈5 kb packaged within a capsid 45–50 nm in diameter and composed of 3 proteins: VP1, VP2, and VP3 (*3*). Twenty members of the polyomavirus family have been identified in mammals and birds (*4*). Four viruses, including the ubiquitous BK and JC viruses, which cause persistent or latent infections, infect humans. Although BK virus, JC virus, and simian virus 40 are tumorigenic in experimental animals and can transform mammalian cells in vitro, no convincing epidemiologic evidence exists for their role in human cancers. We investigated whether patients in France who had MCC carry MCPyV and aimed to identify the strain variations.

## The Study

We conducted our study in 2008 on samples collected during 1991–2008. The study comprised 39 patients with MCC (50–93 years of age, mean 76.9 years; sex ratio 0.95 [19 men, 20 women]). Formalin-fixed and paraffin-embedded (FFPE) tissue specimens from 27 patients and frozen resection specimens from 12 other patients were investigated for MCPyV. In addition, frozen tissue from 8 patients with non-MCC high-grade neuroendocrine carcinomas (5 small-cell lung carcinomas and 3 well-differentiated intestinal carcinomas) and an FFPE tissue specimen from a patient with high-grade neuroendocrine carcinoma of the cervix (human papillomavirus 16 DNA positive) were investigated for MCPyV (43–79 years of age, mean 54.0 years; sex ratio 0.80). All tissue samples were collected for diagnostic purposes, and participants gave written consent in accordance with French ethics regulations.

For DNA preparation from FFPE tissues, 8–10 consecutive sections were subjected to deparaffinization, tissues were then lysed by proteinase K, and DNA was purified by phenol-chloroform extraction. For DNA preparation from frozen specimens, tissue was directly treated with proteinase K and processed as above.

MCPyV was detected by nested PCR by using a first PCR amplification with the LT1 and VP1 primer sets published by Feng et al. (*2*). PCR was performed with 31 cycles for each primer set. A second run of amplification was performed with 2 nested pairs of primers (LT1n, forward 5′-GGCATGCCTGTGAATTAGGA-3′ and reverse 5′-TGTAAGGGGGCTTGCATAAA-3′; and VP1n, forward 5′-TGCAAATCCAGAGGTTCTCC-3′ and reverse 5′-GCAGATGTGGGAGGCAATA-3′) with PCR products from the first round of amplification. Amplification products were subjected to electrophoresis, stained with ethidium bromide, and examined under UV light. To avoid false-negative results from unsuitable DNA quality, a seminested PCR with β-globin primers was run. The first PCR was performed with primers PC04/GH20, and the second PCR used primers PC04/PC03 (*5*). Water was used as PCR-negative controls, and a DNA extract from frozen tissue from an MCC patient was used as a positive control in all experiments. β-Globin amplicons were observed for all frozen MCC tissues investigated (sex ratio 1.0; mean age 71.3 years), whereas only 20 (74%) of 27 FFPE MCC tissues were positive for β-globin by PCR. We found no statistically significant differences in sex ratio and mean age between patients with samples that were FFPE β-globin positive (sex ratio 0.73, mean age 78.5 years) and those that were β-globin negative (sex ratio 0.75; mean age 82.1 years). β-Globin amplicons were detected in all patients with non-MCC neuroendocrine carcinoma (sex ratio 0.86; mean age 60.4 years).

Samples from 21 (66%) of the 32 β-globin–positive MCC patients were PCR positive for MCPyV (Table). All 12 frozen samples of MCC were MCPyV DNA positive, in contrast to FFPE MCC samples in which MCPyV was detected in only 9 (45%) of the 20 investigated. This low level of detection is similar to the 43% reported by Garneski et al. (*6*) and the 54% reported by Ridd et al. (*7*), but lower than the 85% reported by Becker et al. (*8*) in which a smaller DNA segment (80 bp) was amplified by using quantitative PCR. Identity of the PCR products was verified by sequencing or Southern blotting (data not shown). For this purpose, 1 LT1- and 1 VP1-nested PCR products were cloned, sequenced, and used to prepare digoxygenin-labeled probes. VP1 amplicons of ≈350 bp were observed after the first PCR amplification in 9 of the frozen samples from 12 MCC patients. Amplicons of smaller size (≈250 bp) corresponding to a 90-bp deletion in the VP1 open reading frame, as observed by Kassem et al. (*9*) in 1 of 14 patients, were not detected. In contrast, MCPyV DNA was not detected for any of the 9 patients with non-MCC neuroendocrine carcinomas (Table).

**Table:** Detection of Merkel cell polyomavirus by PCR in patients with Merkel cell carcinoma using primers sets within LT and VP gene sequences, France, 2008*

Sample	No. patients	LT1, no. (%)	VP1, no. (%)	Total, no. (%)
Merkel cell carcinoma				
Paraffin-embedded	20	6 (30)	6 (30)	9 (45)
Frozen tissue	12	10 (83)	12 (100)	12 (100)
Other neuroendocrine carcinomas				
Paraffin-embedded	1	0	0	0
Frozen tissue	8	0	0	0

*Non–Merkel cell carcinoma high-grade neuroendocrine carcinomas were 5 small-cell lung carcinomas, 3 well-differentiated intestinal carcinomas, and 1 high-grade neuroendocrine carcinoma of the cervix.

In addition, we investigated the possibility of amplifying the entire VP1 open reading frame by encoding the major capsid protein of MCPyV, using VP1F/VP1R primer sets (5′-CCTGAATTACAAGTAATTGAAGATGGCACC-3′ and 5′-CTGAATAGGAATGCATGAAATAATTCTCAT-3′, respectively). The VP1 gene was amplified from 7 of the frozen samples (Technical Appendix), and 6 of these VP1 amplicons of ≈1,300 bp were cloned and then sequenced. We compared the sequences obtained with the MCPyV sequences from isolates from the United States, Sweden, and Japan (MCC339, EU375804.1; MCC350, EU375803.1; MKL-1, FJ173815; and TKS, FJ464337). The results confirmed the MCPyV VP1 sequence, and only point mutations were observed in the VP1 sequences from the isolates from France compared with the VP1 sequences published (Technical Appendix). The VP1 amino acid sequence from 4 French isolates of MCPyV was identical to that of the Swedish MKL-1 isolate (*10*), and 1 (MKT-23) was identical to that of the MCC339 strain (*2*). MKT-26 showed 2 point mutations that were not reported in any of the other isolates. No French isolate was similar to the U.S. strain MCC350 (*2*), nor to the recently described Japanese isolate (FJ464337). Moreover, 3 silent nucleotide changes were observed in all French isolates, compared with the MCC339 strain, and 1–4 different silent point mutations were observed in isolates MKT-21, MKT-23, MKT-26, MKT-31, and MKT-33.

Because deletions in the viral genome have been reported in the VP2 sequence and the regulatory region of hamster polyomavirus (*11*), a virus that causes lymphomas, the sequence encompassing part of the VP2 protein and the regulatory region (4,876–238) of 7 MCPyV isolates were PCR amplified with the primer set RegF/RegR (5′-TGTTCAGCTGTGAACCCAAG-3′ and 5′-GAGCCTCTCTTTCTTTCCTATTT-3′, respectively), cloned, and sequenced. The N-terminal part of the VP2 of the French isolates was similar to those of the MCC339 U.S. strain and the MKL-1 Swedish strain and differed by 1 amino acid (E41D) from that of the MCC350 U.S. strain. Only minor nucleotide changes were observed within the regulatory region in comparison with the MCC339 strain, except for a deletion of 5 bp (5022–5026) in 5 of the 7 French isolates (Technical Appendix). This deletion has been reported in the MCC350 U.S. strain and the MKL-1 strain. The MKT-23 isolate was similar in the VP1, VP2, and regulatory region to the MCC350 strain.

## Conclusions

Our study confirms the association of MCPyV with MCC (*2*,*6*,*8*,*9*,*12*). However, the primer sets used were not effective for detecting MCPyV DNA in FFPE tissues. In contrast, frozen tissues from MCC patients were all PCR positive, and the entire VP1 gene was easily amplified in 7 of 12 MCC tissues. Our findings demonstrate that strains circulating in Europe are highly conserved and relatively similar to the MCC339 strain in the United States and the MKL-1 isolate from Sweden, suggesting this virus is genetically stable. However, the VP1 sequence of these isolates is relatively different from the VP1 sequence of the MCC350 strain identified in the United States. The MKT-26 VP1 sequence, isolated from an 80-year-old MCC patient, differed from all other isolates.

MCC represents a promising direction for future studies. The MCPyV life cycle needs to be characterized and a greater understanding reached of the natural history of MCPyV infection in humans, including determination of whether MCPyV is associated with other human diseases or malignancies.

## Supplementary Material

Technical Appendix Merkel Cell Polyomavirus Strains in Patients with Merkel Cell Carcinoma
