# Supplementary material for: Merkel Cell Polyomavirus Strains in Patients with Merkel Cell Carcinoma
Source: Emerg Infect Dis. 2009 Jun;15(6):960–2. doi: 10.3201/eid1506.081463 (PMC2727338; doi:10.3201/eid1506.081463)
Supplement: Technical Appendix — Merkel Cell Polyomavirus Strains in Patients with Merkel Cell Carcinoma [file 08-1463_Techapp-s1.pdf]

Merkel Cell Polyomavirus Strains in Patients with Merkel Cell Carcinoma

Technical Appendix

A

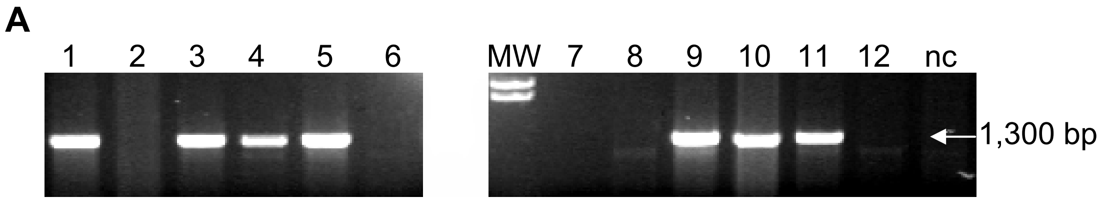

B

VP1 (1–423)

	22	181	185	288	316	362	366	386	422
MCC339	P	D	Q	D	R	Q	D	P	E
MCC350	-	-	E	H	I	-	N	-	Q
MKL-1	-	-	E	-	-	-	-	-	-
TKS	S	-	E	-	-	-	-	L	-
MKT-21	-	-	E	-	-	-	-	-	-
MKT-23	-	-	-	-	-	-	-	-	-
MKT-26	-	G	E	-	-	P	-	-	-
MKT-31	-	-	E	-	-	-	-	-	-
MKT-32	-	-	E	-	-	-	-	-	-
MKT-33	-	-	E	-	-	-	-	-	-

C

Regulatory

MCC339	5013	AAAAAAAGTTGAGTTAAGAGTCTT	5036
MCC350		AAAAAAAGT.....TAAGAGTCTT	
MKL-1		AAAAAAAGT.....TAAGAGTCTT	
TKS		AAAAAAAGTTGAGTTAAGAGTCTT	
MKT-21		AAAAAAAGTTGAGTTAAGAGTCTT	
MKT-22		AAAAAAAGT.....TAAGAGTCTT	
MKT-23		AAAAAAAGTTGAGTTAAGAGTCTT	
MKT-26		AAAAAAAGT.....TAAGAGTCTT	
MKT-31		AAAAAAAGT.....TAAGAGTCTT	
MKT-32		AAAAAAAGT.....TAAGAGTCTT	
MKT-33		AAAAAAAGT.....TAAGAGTCTT	

Technical Appendix Figure. PCR amplification of viral protein (VP) 1 gene in frozen samples. A) Presence of amplicons of ≈1,300 bp in 7 of 12 patients. MW, molecular weight marker; nc, negative control. B) Alignment of VP1 protein amino acid sequences of Merkel cell polyomavirus (MCPyV) isolated from 6 French isolates in comparison with the MCC339 and MCC350 US strains, the MKL-1 Swedish strain (*2,10*), and the TKS Japanese isolate. Sequences were aligned on the amino acid sequence of the MCC339 strain; a hyphen indicates identity. C) Presence or absence of a 5-nt deletion within the regulatory region of MCPyV in 7 isolates from France.
